# Ecological glue for traditional furniture: Optimization of the handicraft for making fish glue

**DOI:** 10.1371/journal.pone.0307974

**Published:** 2024-08-29

**Authors:** Yaqin Qian, Xiangdong Dai

**Affiliations:** 1 Central South University of Forestry and Technology, Changsha, China; 2 Hunan First Normal University, Changsha, China; BOKU: Universitat fur Bodenkultur Wien, AUSTRIA

## Abstract

Through a comprehensive review of published literature on fish glue (FG), the ecological glue for traditional furniture, the traditional handicraft for making FG was found to include six main processes: soaking, steaming, manual smashing, decoction, filtration, and airing. The handicraft that makes FG is manual and is not only time-consuming and laborious but does not have clearly documented standard processes and is thus less repeatable. Considering this, experiments to optimize a new technique for making FG were designed. Six basic technological processes (cutting and drying, crushing, soaking, decoction, filtration, and airing) were investigated to optimize the new glue production technique. The technological processes of the new technique were compared with those of the traditional handicraft method. The results indicated that preparing FG following the optimal processes of the new glue-making technique not only ensured the quality of the glue solution but also outperformed the traditional handicraft technique in the following aspects: 1) it simplifies the production process, reduces labor intensity, and saves time: the soaking time is decreased by 50% and the traditional manual smashing process is not required; 2) it improves the glue yield by 5.42%; and 3) due to introduction of mechanical processing, time and temperature are controllable, rendering production more repeatable and easily up-scaled.

## 1. Introduction

Furniture is an important component of traditional Chinese culture, which represents a rich human cultural heritage stemming from ancient Chinese civilization. Chinese traditional furniture (CTF) has its own style because each individual element carries a deep meaning, is self-contained, and enjoys global popularity [1–4]. The artistic characteristics of CTF in terms of configuration, structure, and decoration all offer rich fountainheads for the development of the contemporary in furniture design and art in China. Particularly, furniture from the Ming and Qing dynasties (1368–1911 AD) is representative of classical CTF either in its texture, craftsmanship, or artistic value. In the handicrafts of CTF, particularly enamel furniture [5], mortise-and-tenon joints and gluing technique are two characteristic traditional handicrafts. Therein, gluing refers to applying a traditional ecological adhesive, namely, fish glue (FG) to mortise and tenon, enamel inlay, and wood splicing [6, 7].

For hundreds of years, FG has always been used as the adhesive in the manufacturing and repair of CTF (especially enamel furniture) because of the following unique functions:

- Tight adhesion; due to the higher strength of FG compared to that of other glues such as pig-skin glue and yak-skin glue [8, 9]
- Firm adhesion; owing to its water absorption, FG stuck in furniture may swell under wet or hot conditions while it shrinking under dry or cold conditions together with furniture, so the gluing is unlikely to loosen and crack.
- FG facilitates later disassembly and repair of furniture [10]. Water absorption of FG is the common weakness of albumen glue. However, once applied to CTF, such weakness becomes advantageous. This is because FG is soluble in hot water [11]. When it becomes necessary to repair and disassemble traditional furniture or replace enameled ornaments, the glued parts of traditional furniture can be wetted with hot water to allow FG to absorb water and soften. This weakens the gluing strength and provides convenience for furniture disassembly while does not damage furniture and enameled ornaments [12, 13].
- It is a green and eco-friendly; FG, as a type of organic albumen glue, does not contain radioactive elements and does not generate noxious gas. When applied in the manufacture of furniture, FG neither damages the furniture nor pollutes the indoor environment [14]. Moreover, swim bladders can be produced repeatedly through aquaculture, so may be classed as an ecological, renewable resource.

Owing to these special benefits arising from the use of FG for CTF manufacture, craftsmen in the past Chinese dynasties attached special importance thereto. According to available literature, the traditional technique used in making FG includes: soaking, steaming, manual smashing, decoction, filtration, and airing [6, 7]. Among them, soaking lasts for about 48 hours in clear water at room temperature. Steaming is used to stew swim bladders in a water bath for 30 minutes while decoction is adopted to heat swim bladders in a water bath for two hours. Thus, FG also has some shortcomings, mainly including the tedious traditional glue-making processes and absence of a convenient and standardized production process to follow. Therefore, while inheriting FG, such a traditional handicraft, it is necessary to explore techniques used to make it. The present study aimed at optimizing the FG-making technique, and can therefore promote the inheritance and development of the traditional handicraft of gluing traditional furniture using FG.

## 2. Methods

### 2.1 Raw materials

Raw materials used in the research mainly included dried swim bladders. Here, the dry swim bladders of wild yellow croaker were selected and purchased online from Derunyuan Food Co., Ltd in Puning City, Guangdong Province, China.

### 2.2 Instruments

A microcomputer screen-display electronic universal testing machine (Ji’nan Liangong Testing Technique Co., Ltd) was used, of which the maximum test force was 200 kN.

A microcomputer-controlled electronic universal testing machine (Shenzhen Sans Material Testing Co., Ltd) was used, of which the maximum test force was 20 kN.

A precision electronic balance (Million Special, Hangzhou Wante Weighing Apparatus Co., Ltd) was used. Its weighing accuracy was ± 0.01 g, it required a 220 V/50 Hz power supply, and its maximum power consumption was 3 W.

A multi-functional crusher (Puji 200-g, Zhejiang Gaoxin Industrial & Trading Co., Ltd) was used. Its capacity was 200 g, it needed a 220 V/50 Hz power supply, and could be operated at the maximum power consumption of 1500 W (28,000 rpm).

A digital thermometer (LCD-110, Hengshui Chuangji Instrument and Apparatus Co., Ltd) was employed; its temperature measurement range was from -50 to 500°C, with an accuracy of ± 1°C, resolution of 0.1°C, and operating ambient temperatures ranging from -30 to 50°C. In addition, the stainless-steel protective tube around each sensor could withstand a pressure of 6.00 MPa, and the digital thermometer was powered by a 1.5 V battery. A digital-display constant-temperature water bath (Changzhou JT Liangyou Instrument Co., Ltd) was used; its temperature was controlled within the range from room temperature to 99.9°C, with the temperature error within ± 1°C. A cast-iron masher (Yongkang Yituo Industry and Trade Co., Ltd) was used. Other devices consisted of glass bottles and stainless-steel containers.

### 2.3 Overall scheme design

The optimal technological processes of the new glue-making technique were screened by optimizing the technique used to make FG. Then, the new glue-making technique was compared with traditional glue-making handicraft to validate the feasibility and value in practice of the new glue-making technique. The research can be divided into two parts, actually into two sub-experiments. Experiment 1 was to optimize the new glue-making technique; on the premise of optimizing the glue-making processes, influences of the decoction duration on the glue yield were assessed and significance analysis of the difference of glue yields under different decoction durations was conducted. Based on Experiment 1, Experiment 2 compared the traditional glue-making handicraft with the new glue-making technique. While comparing the labor intensity, time, and energy cost, the glue yields and gluing strengths of the two were tested. In addition, significance analysis was also performed to evaluate the difference of gluing strengths realized using the two glue-making techniques.

### 2.4 Calculation of the glue yield

The method of measuring the mass of swim bladder residues was utilized to calculate the glue yield; because there were tiny amounts of swim bladder residue left after filtering the glue solution and the water content of these residues was much lower than that of the glue solution, the residues were easily dried. Considering this, the mass of swim bladder residues dried to constant mass was tested at first; then the (constant) mass of FG was ascertained by subtracting the(constant) mass of swim bladder residues from that of tested swim bladders; afterwards, the glue yield was calculated. The calculation formula for the glue yield is deduced as follows (Equation 1).

Glue yield (%) = (dry masses of swim bladders tested in each treatment–dry masses of swim bladder residues in each treatment) ÷ dry masses of swim bladders tested in each treatment × 100% (1)

### 2.5 Test of the gluing strength

Tests were conducted to determine whether the FGs prepared using the two techniques are different in terms of strength or not. The tests were entrusted to Hunan Magic Power Industrial Co., Ltd, who measured two technical indices: tensile shear strength and compressive shear strength.

**Fig 1:**
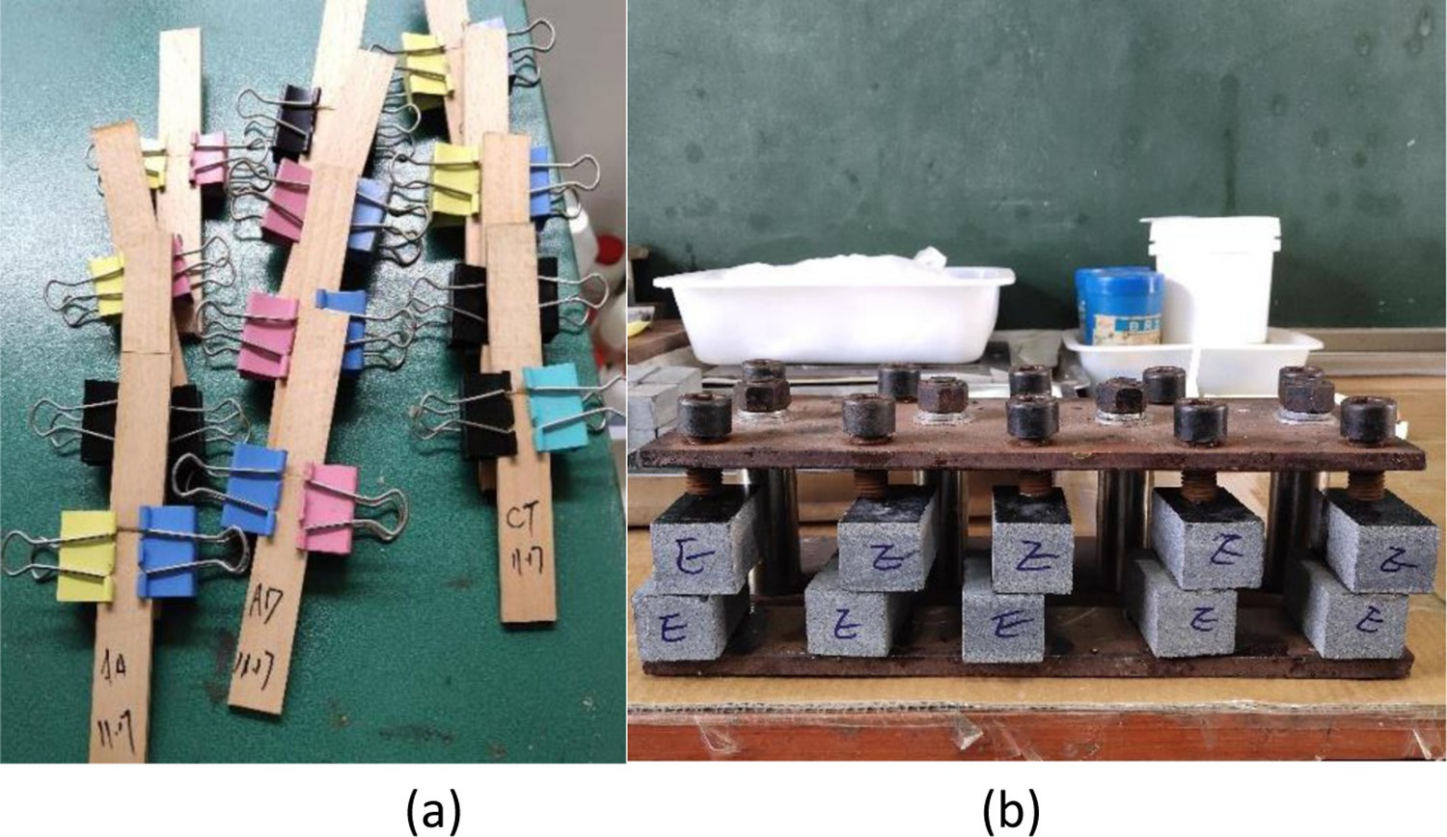
Fixation and drying of specimens for testing the gluing strength (a. Wood specimens; b. Stone specimens).

For tensile shear strength, tests were performed following the test method for tensile shear strength stipulated in GB/T7124-2008 (GB/T7124-1986) [15]. Two types of FG solutions were detected: one was the mixed samples of FG solution obtained in three repetitions using the traditional handicraft (traditional glue); the other was the mixed samples of FG solution obtained in three repetitions using the new technique (new glue). The solid contents (glue concentrations) in the two types of glue solutions were both adjusted to 36%; because FG was used to glue wooden furniture, that is, to glue woods, specimens were made using wood chips (Fig 1A) to test its strength in gluing woods. Each glue solution was used to fabricate five specimens, that is, five repetitions. The specimens were prepared at room temperature (*c*. 22°C) and then naturally aired for six days. The 20-kN electronic universal testing machine was employed for tests.

Compressive shear strength. The compressive shear strength was tested following the test method recommended in GB/T 9966.1–2001. Two types of FG solutions could be detected: one was the mixed samples of FG solution obtained in three repetitions using the traditional technique (traditional glue); the other comprised mixed samples of FG solution obtained in three repetitions using the new technique (new glue). The solid contents (glue concentrations) in the two types of FG solutions were both adjusted to 36%. The specimens were first prepared using woods; however, because all wood specimens were damaged when measuring the compressive shear strength, such that the bond strength failed to be measured, stone was adopted to prepare the specimens. The specimens for testing the compressive shear strength were all prepared using granite (Fig 1B). Five specimens were prepared for each type of glue solution, that is, five repetitions. The room temperature during specimen preparation was 22°C and the prepared specimens were naturally aired for six days. The 200-kN electronic universal testing machine was adopted.

### 2.6 Experiment 1: Optimization of the new glue-making technique

Although traditional glue-making has a long history, the purest form of this handicraft is time-consuming and laborious. As the saying goes, a strong man cannot smash a sliver (150 g) of swim bladders [6, 7]. Meanwhile, the quality of smashed swim bladders cannot be guaranteed due to the high labor intensity, which directly affects the glue yield. Therefore, the optimization experiment for the new glue-making technique was devised to omit the manual smashing process in traditional glue-making, by replacing manual smashing with mechanical crushing; in addition, the automatic digital-display constant-temperature water bath was used to replace the traditionally used steamer in the decoction process. This aimed to (i) reduce the labor intensity; (ii) improve the repeatability of the technique to make FG; (iii) ensure the quality of FG; and (iv) increase the glue yield.

#### 2.6.1 Experimental design

The new glue-making technique was designed to involve six basic technological processes, including cutting and drying, crushing, soaking, decoction, filtration, and airing (drying). According to analysis of relevant data, factors influencing the yield of FG mainly include the species of swim bladders, decoction temperature, and decoction duration. The species and decoction temperature of swim bladders have been introduced in previous research. It has been explicitly proposed that swim bladders of wild yellow croaker (*Larimichthys crocea*) are optimal, and the decoction temperature of swim bladders is that corresponding to micro-decoction (98°C) during water-isolation heating. Therefore, the species and decoction temperature of swim bladders were not treated when designing the experiments, while only the decoction duration was varied. That is, the single-factor experiments of decoction duration were designed, expecting to screen the optimal decoction duration for the new glue-making technique.

The decoction duration was designed at six different levels from 30 min, from which each treatment was prolonged by 30 min. These included the six treatment levels of A (30 min), B (60 min), C (90 min), D (120 min), E (150 min), and F (180 min), each of which was repeated three times, giving a total of 18 treatments (Table 1). Other influencing factors were set the same. The specimens in each treatment were swim bladders of wild yellow croaker from the same brand and with the same mass (20 g). The swim bladders were soaked for the same time (24 h) in the same water (tap water) at the same water temperature (room temperature, about 15°C in the experiments). During decoction, the water temperature in the water bath also remained same (*i*.*e*. the temperature at which micro-decoction occurred at about 98°C).

**Table 1:** Design of experimental treatment levels.

Number of repetitions	Decoction duration					
	A (30 min)	B (60 min)	C (90 min)	D (120 min)	E (150 min)	F (180 min)
1	A1	B1	C1	D1	E1	F1
2	A2	B2	C2	D2	E2	F2
3	A3	B3	C3	D3	E3	F3

#### 2.6.2 Experimental process (glue-making process of the new technique)

The glue-making process of the new technique can be summarized as the following three main steps:

Step 1: Crushing. Dry swim bladders (450 g) were weighed, cut into pieces, and dried to constant mass in the water bath.

**Fig 2:**
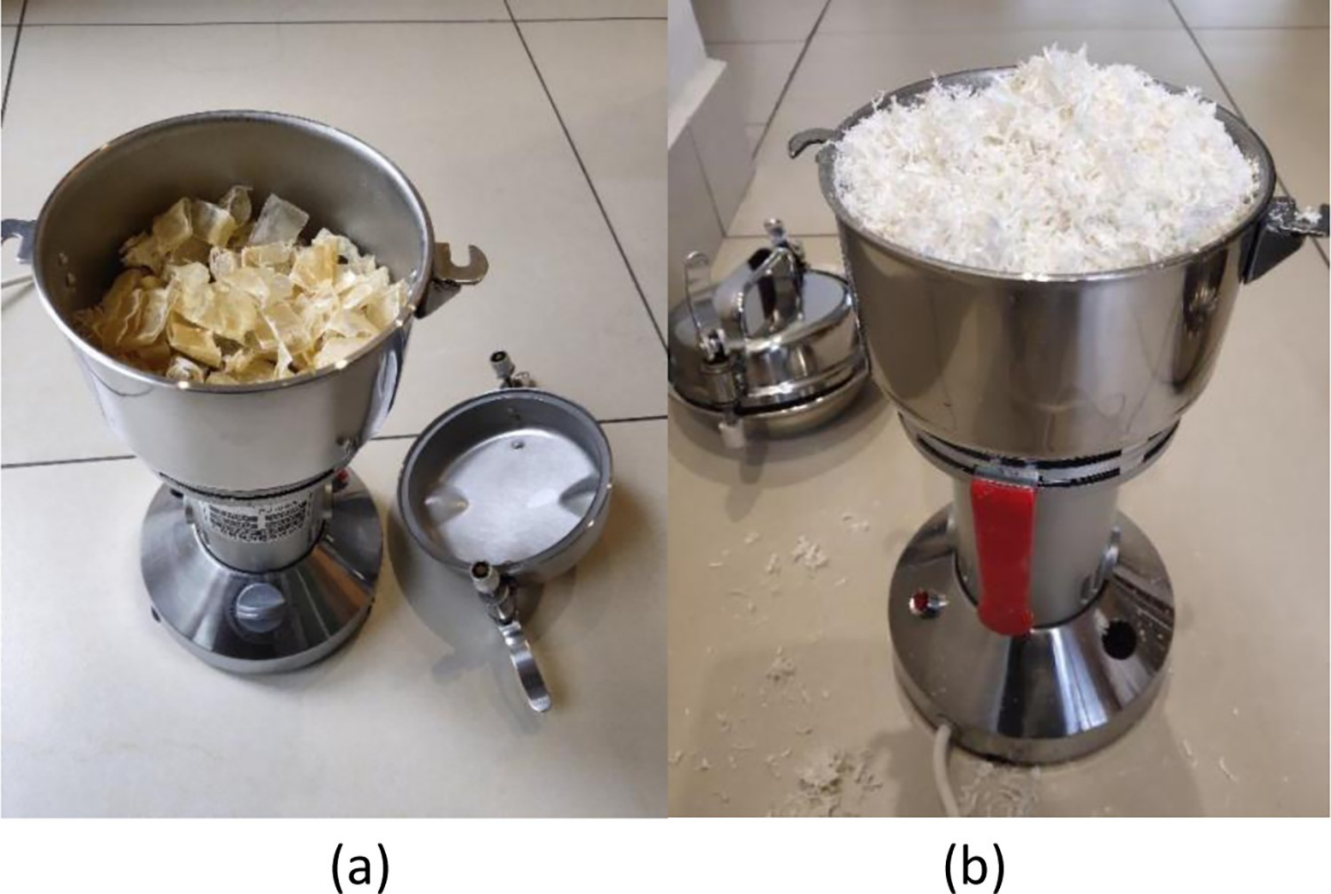
Crushing the dried swim bladders (a. Before crushing; b. After crushing).

A multifunctional miniature crusher was used to crush the dried swim bladders; because it was a miniature crusher, the swim bladders needed to be crushed in batches. The dried swim bladder pieces were placed in the multifunctional miniature crusher and then crushed (Fig 2A). The crusher was run for one minute, then stopped to allow stirring of the swim bladders; the second stage of crushing also lasted for one minute and the swim bladders were stirred after stopping the crusher. In this way, the crusher was started five times and the total crushing time was about five minutes. As a result, the swim bladders appeared like dried meat floss (Fig 2B). Although this failed to crush swim bladders completely into powder, the crushed swim bladders were much finer than the manually smashed ones in the traditional handicraft and were uniform, and favorable for decoction. The crushed swim bladders were placed in a ground-glass stoppered flask and covered for later use.

Step 2: Soaking. According to the different decoction durations in each experimental treatment, the crushed swim bladder samples were weighed in batches. A total of 20 g of material was weighed for each treatment. The samples were soaked in batches at the room temperature (the water temperature during the experiments was about 15°C) for 24 h. The crushed swim bladders were placed in a stainless-steel bowl and slightly compacted to reduce the depth thereof in the bowl. Then, a certain amount of clear water (top water) could be added until the swim bladders were just submerged.

**Fig 3:**
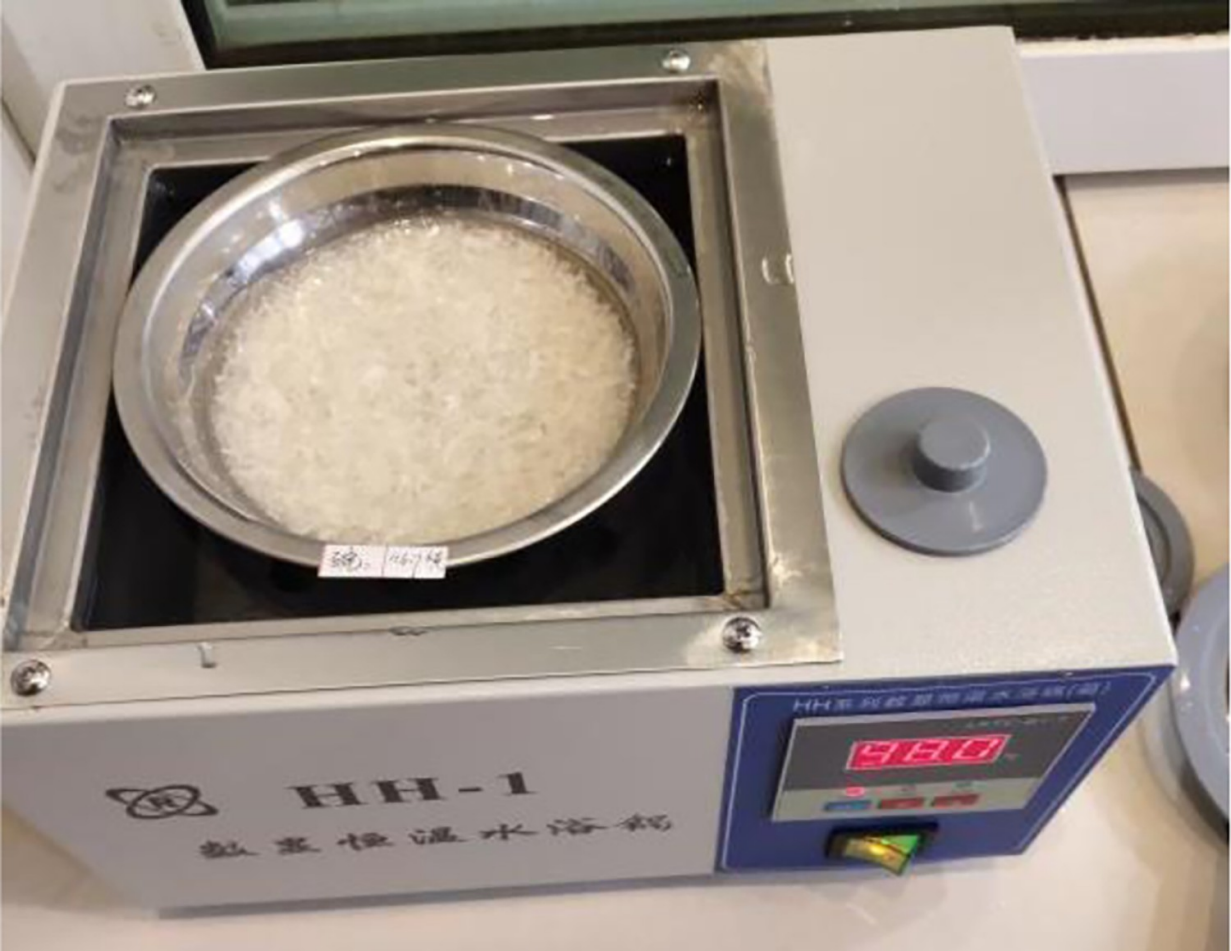
Decoction of swim bladders in the automatic digital-display constant-temperature water bath.

Step 3: Decoction. The pre-soaked swim bladders were decocted in batches according to the decoction durations designed in the experiments. The automatic digital-display constant-temperature water bath was used to heat and decoct the swim bladders. In the process, clear water was poured into the automatic digital-display constant-temperature water bath to two-thirds of the height of the inner wall of the water tank. Then, the water bath was switched on and the upper limit of the temperature was set to 98°C. After the water temperature reached 98°C, the stainless-steel bowl containing soaked swim bladders was placed on the water surface in the water bath (Fig 3) to be heated. In the decoction process, the temperature of swim bladders in the stainless-steel bowl ranged between 50 and 60°C.

After reaching the designed decoction duration, the glue solution was poured out from the water bath and filtered. A same gauze mesh bag was used in each treatment (equivalent to a 60-mesh screen) to filter the glue solution. As the filtered glue solution was not used immediately, the FG (after setting, yet before hardening) was cut into strips with a knife and then aired [10]; the aired glue was preserved for later use. When later used, the dry glue only needed to be soaked in water for 24 h to dissolve as a glue solution by heating in the water bath. During dissolution, the water temperature in the bath should be kept at about 70°C, and the dry glue can be completely dissolved after 10 min.

### 2.7 Experiment 2: Comparison of traditional glue-making handicraft with the new technique

The optimal processes of the new glue-making technique were determined by conducting Experiment 1 (optimization of the new glue-making technique). To verify advantages and disadvantages of the new technique, a comparative experiment for the traditional glue-making handicraft with the new glue-making technique was designed.

#### 2.7.1 Experimental design

The processes of the two glue-making methods are summarized in Table 2. To enhance the comparability of the experiment and reduce the experimental error, the following aspects remained consistent: the quality of swim bladders (wild yellow croaker) used in the two glue-making methods, sample mass used in each time (swim bladders (50 g), which were dried to the constant mass of 44.86 g), water quality for soaking swim bladders (tap water), water temperature (room temperature, about 15°C), decoction duration (120 min), decoction temperature (in a water bath at 98°C and a glue solution temperature of 50 to 60°C), and number of repetitions (three repetitions).

**Table 2:** Design for comparing the technological processes for preparing FG.

Processes of the traditional glue-making handicraft A	Processes of the new glue-making technique B
① Soaking. Swim bladders were cut into pieces and soaked in clear water for 48 h at room temperature.	① Cutting and drying. Swim bladders were cut into pieces and dried in the water bath.
② Steaming. The soaked swim bladders were steamed for 30 min.	② Crushing. The miniature crusher was applied to crush dried swim bladders to a state like dried meat floss.
③ Manual smashing. An iron mortar was used to manually smash the steamed swim bladders to a state that allowed them to be pulled into filaments.	③ Soaking. Swim bladders like dried meat floss were soaked in clear water for 24 h at the room temperature.
④ Decoction. The swim bladders were heated and decocted in a steamer as a water bath for 120 min.	④ Decoction. An automatic digital-display constant-temperature water bath was used for heating and decocting swim bladders for 120 min.
⑤ Filtration. The glue solution was filtered using a gauze mesh bag (60 mesh).	⑤ Filtration. The glue solution was filtered using a gauze mesh bag (60 mesh).
⑥ Airing. The obtained FG solution was aired for later use.	⑥Airing. The obtained FG solution was aired for later use.

#### 2.7.2 Experimental process (glue-making processes used in the two techniques)

The glue-making process involves two parts: one is to prepare FG using the traditional handicraft and the other is to fabricate FG using the new technique.

*2*.*7*.*2*.*1 Glue-making process in the traditional handicraft*. The processes of making FG using the traditional handicraft completely followed the “processes of the traditional glue-making handicraft A” in Table 2. The specific glue-making steps are summarized as follows:

- **Soaking.** Some 50 g of selected dry swim bladders were weighed (dried to constant mass of 44.86 g), cut into pieces with a pair of scissors, and placed in the stainless-steel bowl, to which clear water was added to submerge the dried swim bladders. The soaking lasted for 48 h at room temperature. After soaking for 24 h, swim bladders were observed to ascertain whether there were any hard cores remaining and incomplete softening. After soaking for 48 h, when no hard cores could be seen and the specimen felt slippery and soft when pinching the swim bladders, the soaking was deemed complete.
- **Steaming.** The soaked swim bladders were put in the stainless-steel bowl, to which a little clear water was added (water was visible while not completely submerging the swim bladders). Then, the stainless-steel bowl was placed in the steamer to be heated and steamed. The steaming duration was about 30 min, or until the swim bladders became soft and transparent.
- **Manual smashing.** The steamed hot swim bladders were transferred to an iron masher (iron mortar and pestle) and then manually smashed. The manual smashing duration depended on the smashing effect, as well as the mass of swim bladders manually smashed in each time and upon the patience of the craftsmen. Generally, it took 2 to 2.5 h for a craftsman to manually smash 100 g of swim bladders, and the time could be 3 to 3.5 h for a craftswoman. Swim bladders were regarded as successfully smashed provided they became starchy and formed fine filaments when being picked up using the iron pestle.
- **Decoction.** The swim bladders manually smashed into a starchy mass were shoveled into the stainless-steel bowl, and a small amount of hot water (until the smashed swim bladders were submerged) was added. Then, the stainless-steel bowl was put in the steamer to heat the swim bladders. Water in the steamer was boiled, then the input power was decreased to a simmer [16]. The temperature was 98°C at the simmer and the temperature of glue solution in the stainless-steel bowl during decoction was about 50 to 60°C. The water level in the steamer needed to be monitored during decoction and boiling water should be added timeously. Meanwhile, the swim bladders should be stirred once or twice with a pair of stainless-steel chopsticks to facilitate the uniform dissolution of the glue. After being decocted for about 2 h (120 min), the swim bladders were deemed to have been decocted when they were completely dissolved, and only small amounts of egg-drop-shaped materials floated on the glue solution during stirring [16].
- **Filtration.** A gauze mesh bag (equivalent to a 60-mesh screen) and a stainless-steel bowl were adopted. At first, after opening the gauze mesh bag in the stainless-steel bowl, the decocted FG solution was poured into the gauze mesh bag (wearing rubber gloves to prevent empyrosis). The bag mouth was then tightened, and the bag squeezed from top to bottom (by hand) to extrude the FG solution into the stainless-steel bowl. After filtering undissolved substances floating in the glue solution, the FG solution was obtained. The FG solution squeezed in the bowl could be used normally. If the FG solution was too thin to use, the bowl could be heated in the steamer to evaporate some of the water and concentrate the solution [8].
- **Airing.** If the FG solution was not used immediately, it was sliced into strips after setting (yet before hardening), aired, and preserved for later use [7]. When next used, the dry glue only needed to be soaked in clear water for 24 h, then heated to form a glue solution in a water bath [17].

*2*.*7*.*2*.*2 Glue-making processes in the new technique*. The processes of making FG using the new technique completely followed the “processes of the new glue-making technique B” in Table 2. The glue-making processes are completely consistent with “2.6.2 Experimental process (glue-making process of the new technique)” in Experiment 1.

## 3. Results

### 3.1 Difference of glue yields under different decoction durations

The masses of swim bladder residues dried to the constant masses in six treatments in Experiment 1 (Optimization of the new glue-making technique) were measured using the aforementioned method of calculation of the glue yield (Table 3).

**Table 3:** Dry masses of swim bladder residues in each experimental treatment (unit: g).

Number of repetitions	A (30 min)	B (60 min)	C (90 min)	D (120 min)	E (150 min)	F (180 min)
1	7.22	5.38	3.14	2.95	3.2	2.81
2	6.91	6.01	3.57	3.19	2.81	3.02
3	7.02	5.89	3.61	3.04	3.08	2.66
**Mean**	**7.05**	**5.76**	**3.44**	**3.06**	**3.03**	**2.83**

The average dry masses of FG solutions obtained in each treatment were then deduced to be 12.95, 14.24, 16.56, 16.94, 16.97, and 17.17 g according to the average dry masses of swim bladder residues in each treatment. Afterwards, average glue yields of each treatment were calculated to be 64.75%, 71.2%, 82.8%, 84.7%, 84.85%, and 85.85% according to the dry masses of glue solutions.

**Fig 4:**
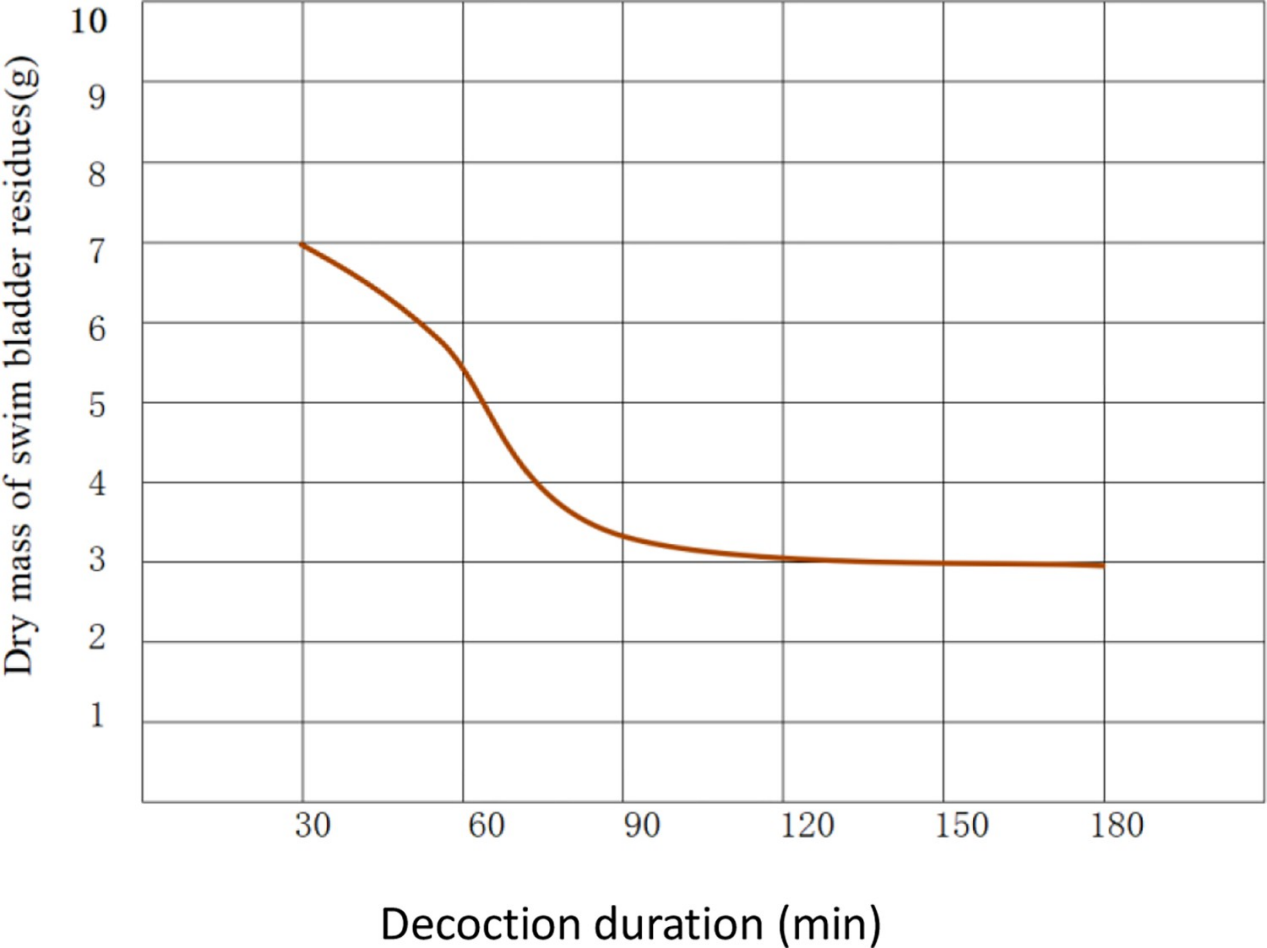
Changes in the dry mass of swim bladder residues.

The experimental results show that as the decoction duration in each treatment prolonged, the mass of swim bladder residues left after decoction decreased, that is, the (dry) mass of the residues was inversely proportional to the decoction duration. The changes in the dry mass of swim bladder residues are illustrated in Fig 4. Whereas, when the decoction duration reached 90 min, the decrease in the mass of the residues reduced: after decoction for 120 min, the decrease became quasi-stable.

**Fig 5:**
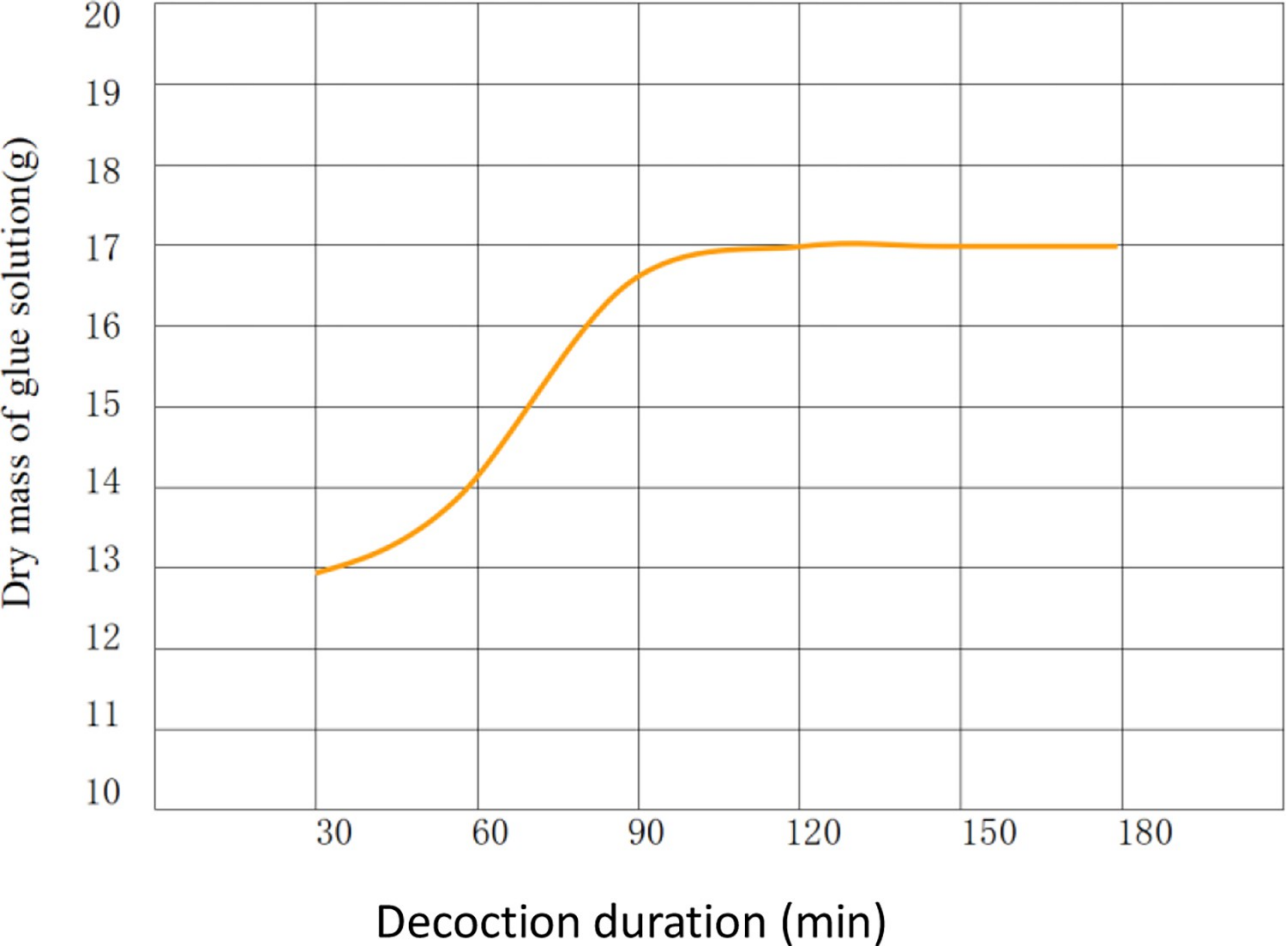
Changes in the dry mass of glue solution.

On the contrary, the mass of obtained glue solution increased with the prolonged decoction duration, that is, the mass of glue solution was directly proportional to the decoction duration. The changes in the (dry) mass of glue solution are shown in Fig 5.

**Fig 6:**
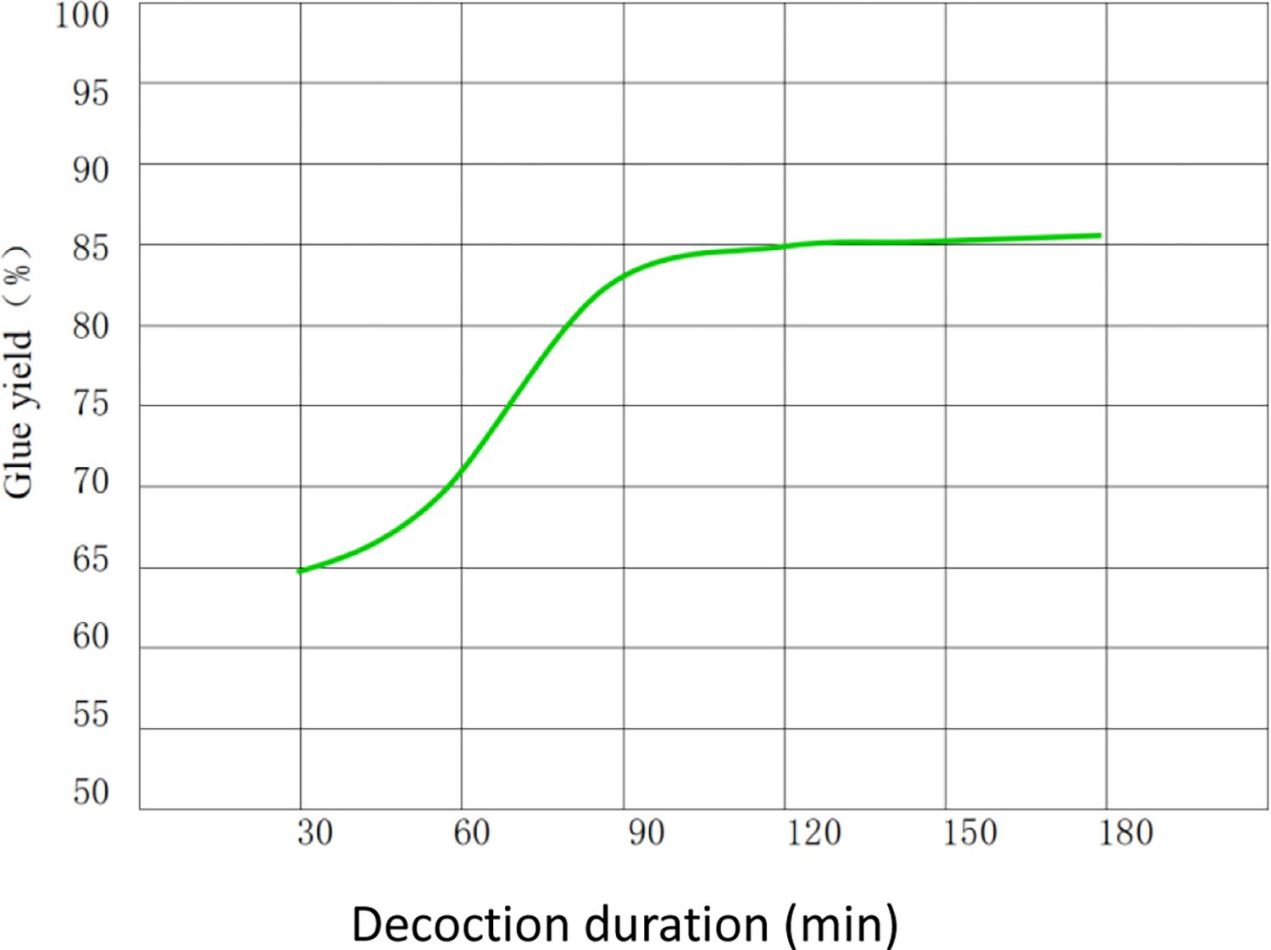
Changes in the glue yield.

The glue yield changed in the same trend as the mass of glue solution, that is, it was directly proportional to the decoction duration. The changes in glue yield are illustrated in Fig 6.

Through significance analysis (analysis of variance) of the difference of the glue yields in each treatment in Experiment 1, the glue yield of treatment D (120 min) differs insignificantly from that of treatment F (180 min) with the highest glue yield. However, treatment D saves about one-third of the decoction time compared with treatment F and therefore its power consumption is also reduced by about one-third compared with that of treatment F. Although treatment C (90 min) differs insignificantly from treatment D in terms of the glue yield, it shows significant differences with treatment F, reaching an *α*-level of 0.01. Therefore, analysis of the experimental results indicates that treatment D is optimal with the highest efficiency ratio after comprehensive analysis and evaluation of the glue yield, time cost of decoction, and power consumption. In the experiment to optimize the new glue-making technique designed in Experiment 1, 120 min (treatment D) is deemed to be the optimal decoction duration. That is to say, those technological processes of treatment D are optimal ones as optimized by Experiment 1. This can be summarized as the following six processes:

- **Cutting and drying.** The dry swim bladders were cut into pieces with a pair of scissors, and then dried in the water bath, or aired, which aimed to decrease the water content thereof and make swim bladders easier to crush.
- **Crushing.** The multifunctional miniature crusher was adopted to crush the dry swim bladders to a state akin to dried meat floss. During the crushing, the crusher was run for one minute for each time, and then the swim bladders were stirred after each crushing stage. This was repeated five times, and the crusher was run for a total of five minutes.
- **Soaking.** The crushed swim bladders were completely submerged in clear water in the stainless-steel bowl for 24 h at room temperature.
- **Decoction.** The swim bladders were decocted in the water bath using the constant-temperature water bath, in which the water temperature was set to 98°C while continuously heating the swim bladders for 120 min. Two points should be noted in this process: the swim bladders should be stirred once to twice and boiling water needed to be added to the water bath timeously.
- **Filtration.** A gauze mesh bag (equivalent to a 60-mesh screen) was utilized to filter the decocted glue solution to remove residues.
- **Airing.** If the prepared glue solution was not used immediately, it was sliced into strips with a knife after initial setting (but before hardening), then aired for later use. When next used, the dry glue could be soaked in clear water for 24 h and then dissolved by heating in the water bath. In the process, the water should be kept at about 70°C, at which the FG could be completely dissolved within about 10 min.

### 3.2 Comparison of glue yields of the two glue-making techniques

Likewise, the dry mass of swim bladder residues, the dry mass of FG, and glue yield in the traditional glue-making handicraft in Experiment 2 were measured according to the above calculation method of the glue yield, as displayed in Table 4; those in the new glue-making technique in Experiment 2 are listed in Table 5.

**Table 4:** Glue yields in the traditional handicraft.

Number of repetitions	Dry mass of residues (g)	Dry mass of FG (g)	Glue yield (%)	Dry mass of tested swim bladders (g)
A1 (repetition 1)	8.40	36.46	81.28	44.86
A2 (repetition 2)	8.06	36.80	82.03	44.86
A3 (repetition 3)	8.59	36.27	80.85	44.86
**Mean**	**8.35**	**36.51**	**81.39**	

**Table 5:** Glue yields in the new technique.

Number of repetitions	Dry mass of residues (g)	Dry mass of FG (g)	Glue yield (%)	Dry mass of tested swim bladders (g)
B1 (repetition 1)	6.27	38.59	86.02	44.86
B2 (repetition 2)	6.81	38.05	84.82	44.86
B3 (repetition 3)	6.03	38.83	86.56	44.86
**Mean**	**6.37**	**38.49**	**85.80**	

**Fig 7:**
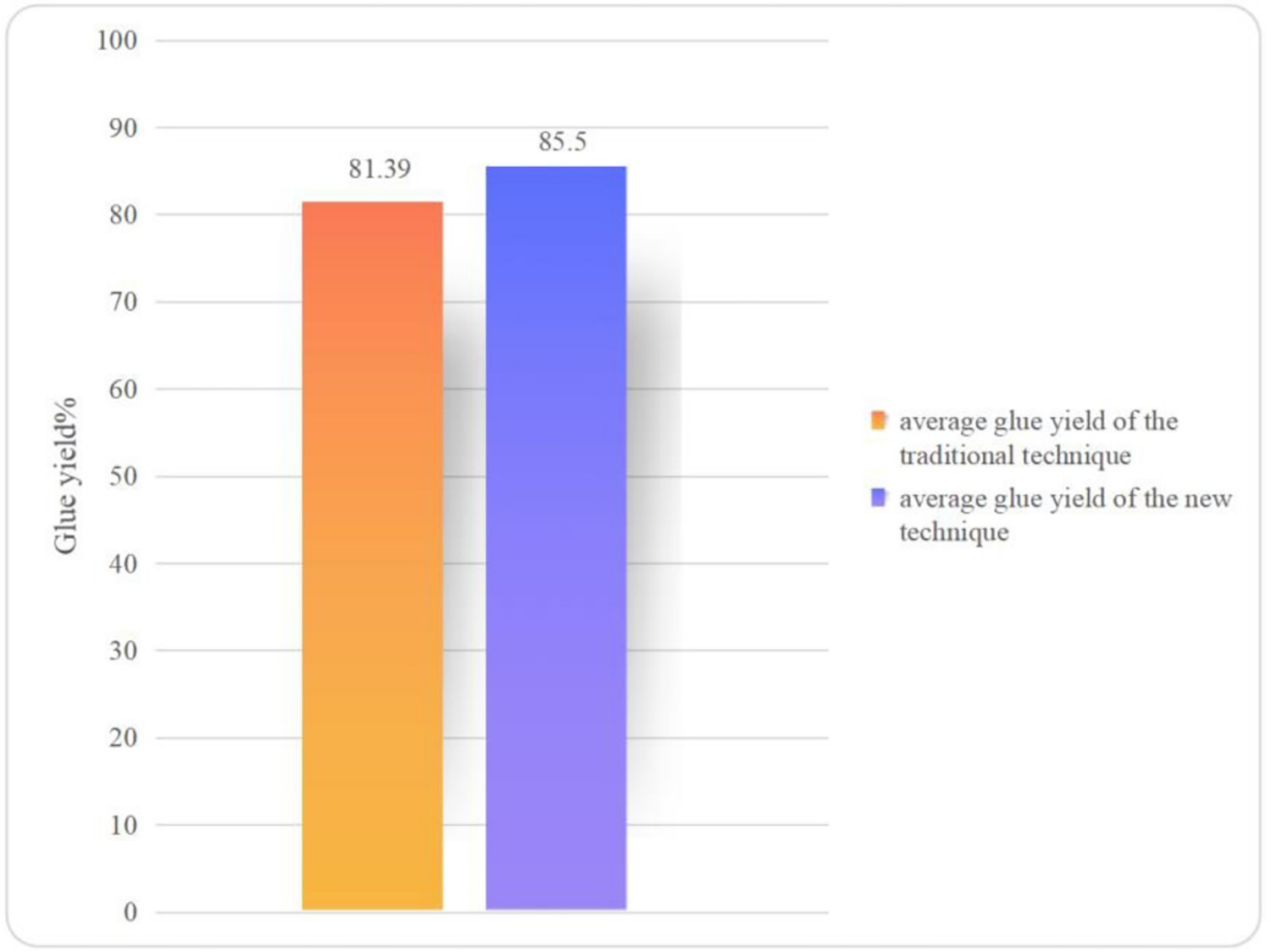
Comparison of glue yields.

It can be seen from Table 4 that the average glue yield (over three repetitions) is 81.39% in the glue-making results using the traditional handicraft, while that in the glue-making results using the new technique (Table 5) is 85.80%. The average glue yield in the new technique is 4.41 percentage points higher than that in the traditional handicraft, that is, the glue yield increases by 5.42%. Such a difference can also be obviously seen in Fig 7. The experiment proves that the glue yield can be significantly improved when using the new technique to make FG.

### 3.3 Comparison of gluing strengths of the two glue-making techniques

The tensile shear strengths of the two types of FG prepared using the two glue-making techniques were tested using the aforementioned method (Table 6).

**Table 6:** Tensile shear strengths of the two types of FG (unit: MPa).

FG specimens	Solid contents of glue solution	Specimen 1	Specimen 2	Specimen 3	Specimen 4	Specimen 5	Mean
Traditional glue	36%	5.26	5.70	9.37	9.43	8.40	**7.632**
New glue	36%	8.26	8.23	7.13	8.87	5.93	**7.684**

Significance analysis (analysis of variance) of the difference in tensile shear strengths of the two types of FG indicates that *F* (0.0025) < *F*_0.05_ (5.32), which indicates a lack of significance and obviates the need to conduct multiple comparisons between mean values. This finding suggests that the traditional handicraft and new technique do not lead to significant differences in the tensile shear strengths.

The compressive shear strengths of the two types of FG prepared using the two glue-making techniques were tested using the above method, as listed in Table 7.

**Table 7:** Compressive shear strengths of the two types of FG (unit: MPa).

FG specimens	Solid contents of glue solution	Specimen 1	Specimen 2	Specimen 3	Specimen 4	Specimen 5	Mean
Traditional glue	36%	4.849	3.845	3.935	4.846	4.303	**4.3556**
New glue	36%	3.259	4.769	3.796	5.302	4.566	**4.3384**

Note: The specimens were made of glued stone, so their measured strengths were lower than those of glued wood [18].

Through significance analysis of the difference in compressive shear strengths of the two types of FG, it is obtained that *F* (0.0016) < *F*_0.05_ (5.32), implying insignificance and obviating the need to perform multiple comparisons between mean values. This finding indicates that the traditional handicraft and new technique do not lead to significant differences in the compressive shear strengths.

## 4. Discussion

### 4.1 Analysis of the labor intensity and time required for making glue

**Fig 8:**
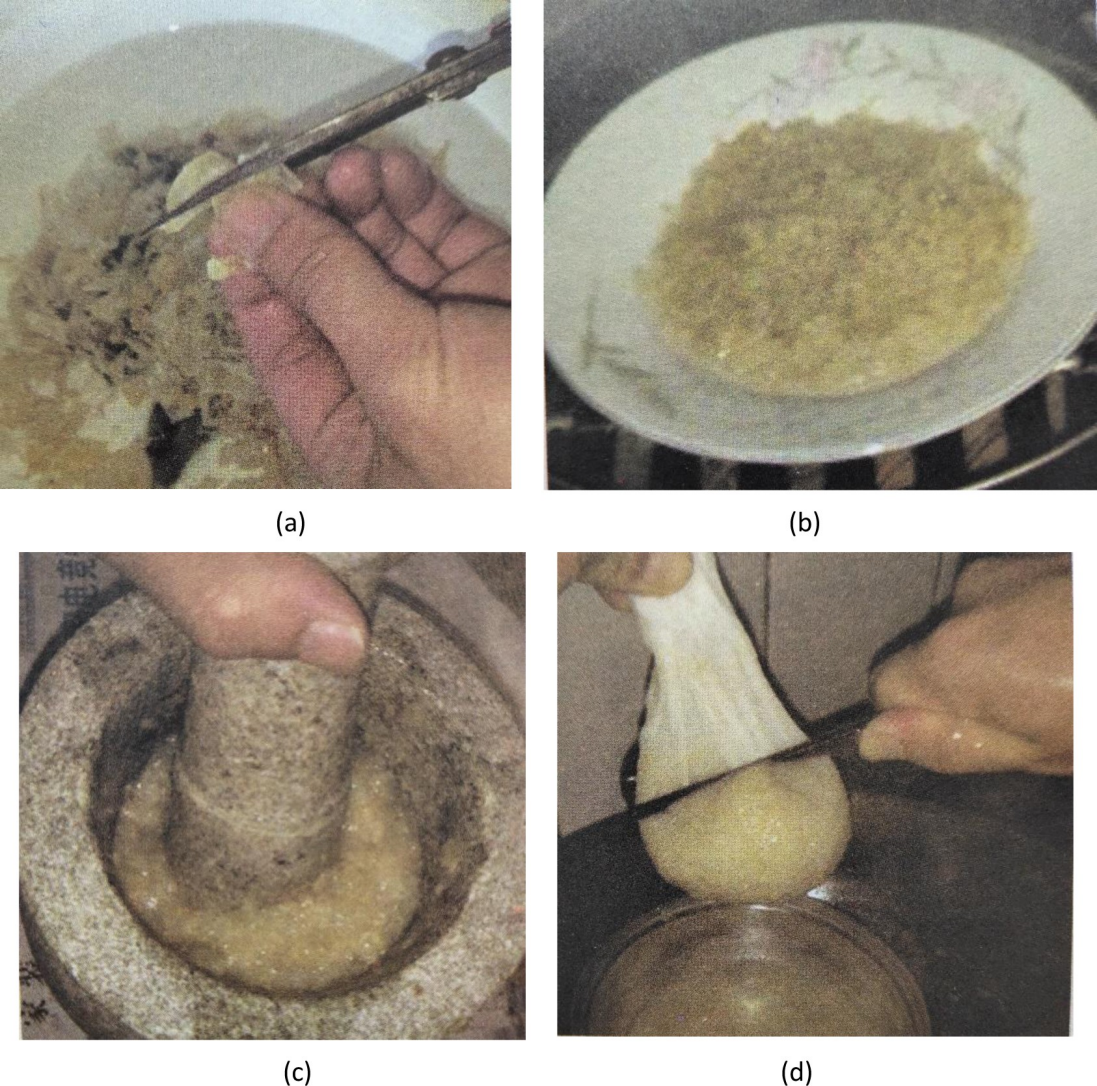
Preparation of FG using the traditional method (a. cutting and soaking; b. steaming; c. manual smashing; d. decoction and then filtering) [6].

**Fig 9:**
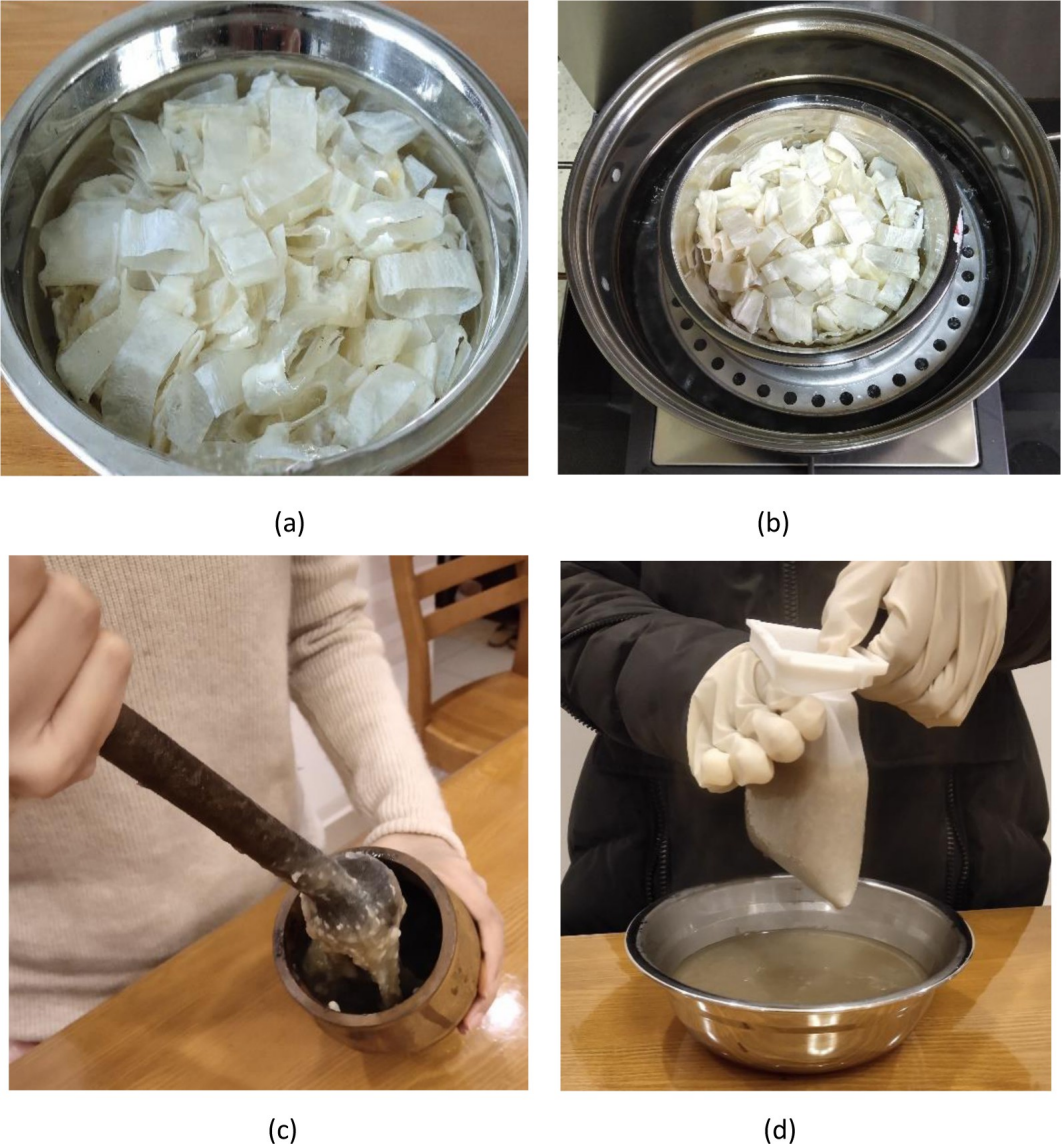
Reproduction of the traditional glue-making handicraft (a. cutting and soaking; b. steaming; c. manual smashing; d. decoction and then filtering).

FG, the glue used for traditional furniture, is an eco-friendly glue. It has always been prepared using the traditional glue-making method, referred to as the “glue-making method by manually smashing swim bladders”, to which improvements have not been reported. For example, the method for making FG introduced by engineer Lu Yuzhang [7] is also the “glue-making method by manually smashing swim bladders”. Dr. Duan Zhenhua [17] introduced different methods for processing swim bladders as food, medicine, and glue, in which the method of processing swim bladders as glue is also the “glue-making method by manually smashing swim bladders”. Although many studies have introduced the traditional method of making FG, the one introduced by Dr. Niu Xiaoting [6] is more detailed. The glue-making processes include cutting and soaking, steaming, manual smashing, decoction, filtering, and airing (Fig 8A–8D). To verify the traditional glue-making method, its glue-making process was reproduced (Fig 9A–9D).

By reproducing the traditional glue-making handicraft, it is summarized that: when using the traditional glue-making handicraft to prepare FG, complete swelling of swim bladders (to a transparent, slippery, and soft state) soaked in tap water at the room temperature takes 48 h; steaming takes 30 min; manually smashing 100 g of swim bladders takes 2 to 3 h. Manual smashing is particularly laborious (due to the high viscosity of the substance).

Swim bladders are easily soaked in the new glue-making technique (Figs 2 and 3) because they are crushed before being soaked. A period of 24 h is enough to soak swim bladders, which cuts 50% from the soaking duration compared with the traditional glue-making technique. Two processes, namely, steaming and manual smashing are omitted in the new glue-making technique. Especially manual smashing, it is a time-consuming and laborious process with which difficulties arise in reaching a satisfying effect. Two processes, namely, drying and crushing are added in the new technique. Crushing only takes several minutes and drying can also be omitted. If swim bladders are not dried, they only need to be crushed two more times, each stage of which takes one minute. Therefore, the new glue-making technique saves the time for soaking and steaming, omits manual smashing and greatly reduces the labor intensity.

## 4.2 Repeatability analysis of glue-making techniques

The traditional glue-making handicraft uses the “glue-making method by manually smashing swim bladders”, the key to which is the difficulty in controlling the manual smashing quality. Because manual smashing of swim bladders is laborious and craftsmen differ in the physical power they choose to deliver, it is difficult to control the time taken in manual smashing and there is no standard to follow. The duration can only be determined according to the physical power of the craftsman. That is to say, swim bladders with a mass of 100 g smashed by each craftsman in the same time period may differ in the quality, and it is impossible to smash swim bladders manually into fine filaments of uniform size. For smashed swim bladders of different qualities, the decoction duration also differs, as does the glue yield. In the new glue-making technique, mechanical smashing (for the same duration of 5 min each time) is used to replace manual smashing. In this way, the quality of swim bladders smashed in different batches reaches the same standard and the swim bladders are smashed uniformly each time (Fig 2b). Each batch of swim bladders can therefore be decocted for the same time (120 min), after which a relatively stable glue yield can be obtained. This indicates that FG prepared using the new glue-making technique has standards to follow and the technique is highly repeatable, standardizing the glue-making technique.

### 4.3 Influences of the two glue-making techniques on the quality of FG

**Fig 10:**
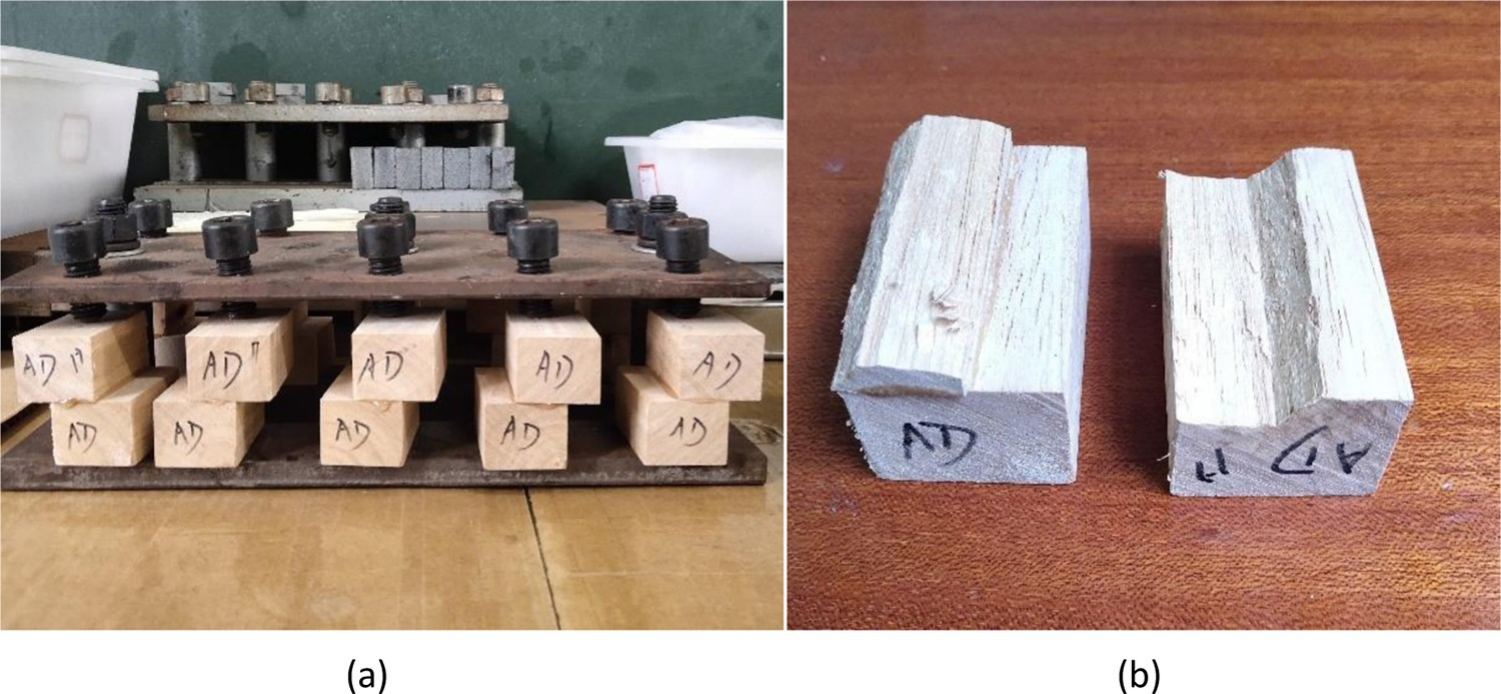
Wood specimens for testing the compressive shear strength (a. prepared wood specimens; b. damaged specimens).

In the present research, the technique for making FG as an adhesive was explored. Therefore, the gluing strength was mainly taken as the quality-evaluation standard for FG prepared using the two glue-making techniques, so as to test whether gluing strengths of FG prepared using the two techniques are significantly different or not. Two indices, namely, the tensile shear strength and the compressive shear strength were selected; because FG was mainly utilized to glue wood pieces in practical production, wood was selected to prepare specimens at first. The tensile shear strength was tested smoothly, and the average tensile shear strengths of FG prepared using the traditional and new glue-making techniques were 7.632 and 7.684 MPa, respectively. Results in Section 3.3 show that tensile shear strengths of the two types of FG did not differ to any significant extent. When testing the compressive shear strength, because the FG was too viscous when used with wood, all wood specimens were damaged (Fig 10A and 10B) and the real compressive shear strength could not be tested. Considering this, granite was used to fabricate specimens (Fig 1B) to retest the compressive shear strength. The average compressive shear strengths of FG prepared using the traditional and new techniques were tested to be 4.3556 and 4.3384 MPa, respectively. This was probably because the viscosity of FG with stone was not as high as that with wood, so that the measured compressive shear strengths were relatively low, however, because the two types of FG were tested using the same stone, the measured compressive shear strengths were also comparable. Results in Section 3.3 show that the two types of FG did not show significant differences in compressive shear strength.

**Fig 11:**
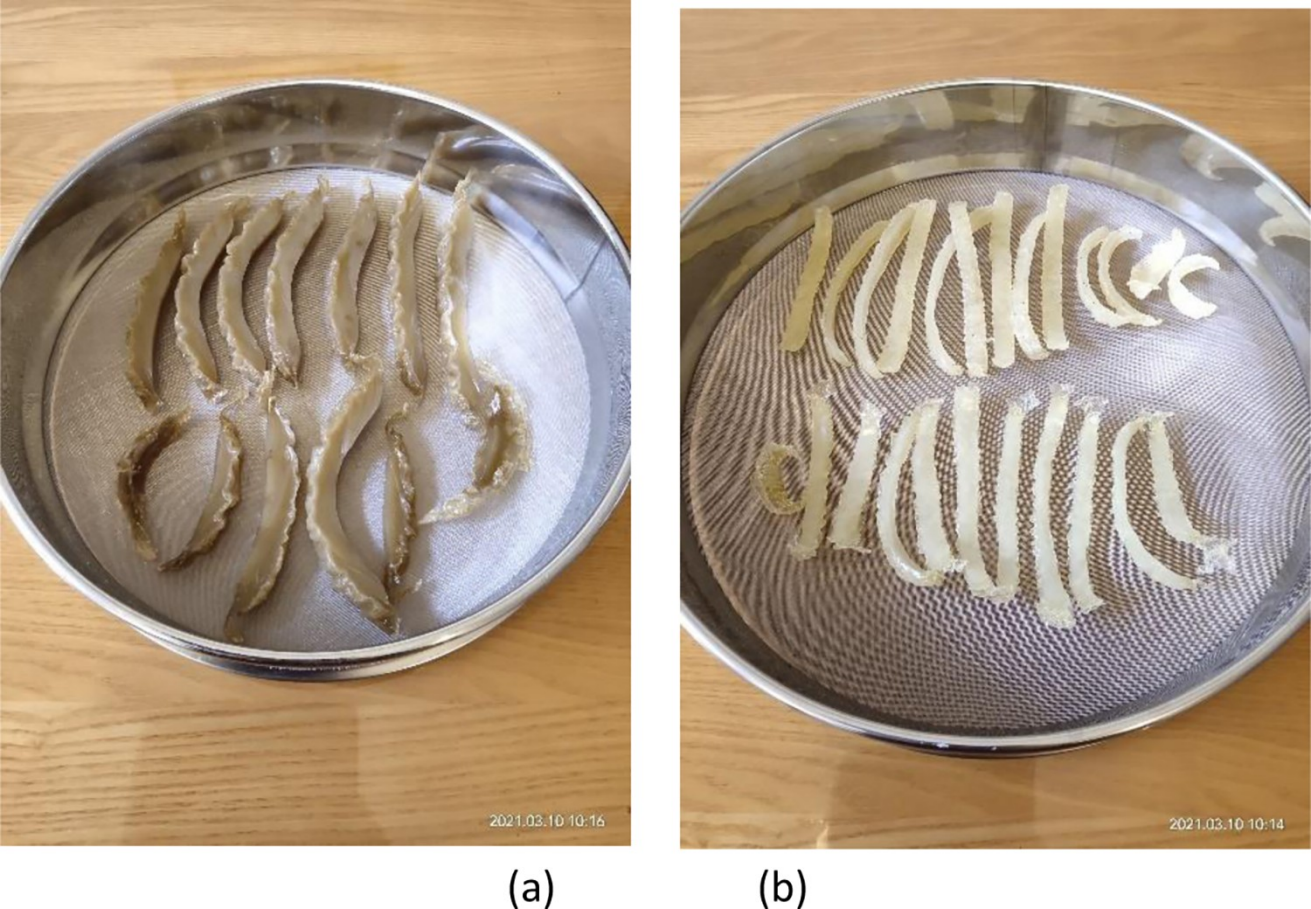
Color comparison of FG prepared using the two techniques (a. FG prepared using the traditional technique; b. FG prepared using the new technique).

Meanwhile, in the traditional glue-making technique, the iron (or stone) mortar and pestle are generally used to manually smash swim bladders. Due to the wearing of the mortar and pestle, FG is, to some extent, polluted. For example, the iron mortar and pestle were applied to manually smash swim bladders when preparing FG using the traditional glue-making technique in Experiment 2 in the research. As a result, the obtained glue solution is the color of mung beans (Fig 11A), much darker than that prepared using the new technique, probably as a result of pollution due to abrasive wear of the mortar and pestle in the manual smashing process. In comparison, the new glue-making technique does not pollute the glue solution, so the prepared glue solution is not different from swim bladders in terms of color (Fig 11B).

The above analysis indicates that the gluing strength of the FG fabricated using the new glue-making technique does not differ significantly from that prepared using the traditional technique. Additionally, the color of FG prepared using the new technique is superior to that fabricated using the traditional one. That is to say, the quality of FG prepared using the new technique is higher than that prepared using the traditional handicraft according to overall evaluation.

### 4.4 Influences of the two glue-making techniques on glue yield

The traditional glue-making handicraft uses the “glue-making method of manually smashing swim bladders”, making it challenging to smash swim bladders into uniform filaments by hand unless doubling the duration of that stage of the process. This, however, increases the labor intensity and prolongs the glue-making time; otherwise, the smashed swim bladders may differ in quality and size, which further leads to different rates of dissolution of the swim bladders during decoction. In the new glue-making technique, mechanical smashing is adopted to crush swim bladders to a state like dried meat floss with the same fineness (Fig 2B). This enables swim bladders to uniformly turn into the glue solution at a faster rate. Therefore, within the same decoction duration (120 min), the average glue yields are separately 81.39% and 85.80% when using the traditional and new glue-making techniques, with the latter being 5.42% higher than the former. Of course, the glue yield can be further improved if the decoction stage of the traditional glue-making technique is further prolonged; however, this not only increases labor cost but also increases energy consumption (including electricity), from which the loss outweighs the gain.

## 5. Conclusion

Chinese traditional furniture, especially that dates from the Ming and Qing dynasties (1368–1911 AD), not only has an attractive appearance but is exquisitely handcrafted. Gluing using ecological FG is one of the traditional handicrafts and is still widely applied to the manufacture of Chinese-style furniture and repair of traditional furniture [19]. However, the traditional method of making FG is tedious, time-consuming, laborious, and less repeatable. To cope with these problems, the traditional glue-making handicraft was optimized through the optimization experiment of the new glue-making technique (Experiment 1), thus exploring a new set of glue-making processes, including cutting and drying, crushing, soaking, decoction, filtration, and airing. The new glue-making technique entails the replacement of manual smashing with mechanical crushing and uses an automatic digital-display constant-temperature water bath to replace the traditional steamer used in the decoction process. As verified by the “comparative experiment of traditional and new glue-making techniques (Experiment 2)”, the gluing strength of FG prepared using the new technique does not differ obviously with that prepared using the traditional handicraft and it meets the standard of traditional FG. That is, the new glue-making technique does not affect the quality of FG. Meanwhile, the new technique is superior to the traditional one: it not only improves the glue yield by 5.42% but also substantially reduces the glue-making time (soaking only lasts for 24 h, which is only 50% of that used traditionally; the onerous manual smashing process is also omitted). Moreover, the new technique also reduces the labor intensity associated with making glue (by omitting the onerous manual smashing process) and improves the repeatability of the glue-making technique. Furthermore, it realizes standardization of the glue-making technique (using mechanical timed crushing and the automatic, constant-temperature water bath for decoction, of which the time and temperature are controllable). The results are significant for actively guiding and promoting the inheritance and application of bonding technology using FG and the improvement of techniques for the production of FG.

## Supporting information

S1 File (DOCX)
