# Supplementary material for: Ecological glue for traditional furniture: Optimization of the handicraft for making fish glue
Source: PLoS One. 2024 Aug 29;19(8):e0307974. doi: 10.1371/journal.pone.0307974 (PMC11361600; doi:10.1371/journal.pone.0307974)
Supplement: S1 File — (DOCX) [file pone.0307974.s001.docx]

SUPPLEMENTARY FILES

2.3.1 Changes in glue yield

The masses of swim bladder residues dried to the constant masses in each treatment are listed in Table 2. The dry masses of fish glue solutions obtained in each treatment were deduced (Table 3). Afterwards, glue yields of each treatment were calculated using the above formula (Table 4).

**Table 2. Dry masses of swim bladder residues in each experimental treatment (unit: g)**

| Number of repetitions | A  (30 min) | B  (60 min) | C  (90 min) | D  (120 min) | E  (150 min) | F  (180 min) |
| --- | --- | --- | --- | --- | --- | --- |
| 1 | 7.22 | 5.38 | 3.14 | 2.95 | 3.2 | 2.81 |
| 2 | 6.91 | 6.01 | 3.57 | 3.19 | 2.81 | 3.02 |
| 3 | 7.02 | 5.89 | 3.61 | 3.04 | 3.08 | 2.66 |
| **Mean** | **7.05** | **5.76** | **3.44** | **3.06** | **3.03** | **2.83** |

**Table 3. Calculated dry masses of fish glue in each treatment (unit: g)**

| Number of repetitions | A  (30 min) | B  (60 min) | C  (90 min) | D  (120 min) | E  (150 min) | F  (180 min) |
| --- | --- | --- | --- | --- | --- | --- |
| 1 | 12.78 | 14.62 | 16.86 | 17.05 | 16.8 | 17.19 |
| 2 | 13.09 | 13.99 | 16.43 | 16.81 | 17.19 | 16.98 |
| 3 | 12.98 | 14.11 | 16.39 | 16.96 | 16.92 | 17.34 |
| **Mean** | **12.95** | **14.24** | **16.56** | **16.94** | **16.97** | **17.17** |

**Table 4. Calculated glue yields in each treatment (unit: %)**

| Number of repetitions | A  (30 min) | B  (60 min) | C  (90 min) | D  (120 min) | E  (150 min) | F  (180 min) |
| --- | --- | --- | --- | --- | --- | --- |
| 1 | 63.9 | 73.1 | 84.3 | 85.25 | 84 | 85.95 |
| 2 | 65.45 | 69.95 | 82.15 | 84.05 | 85.95 | 84.9 |
| 3 | 64.9 | 70.55 | 81.95 | 84.8 | 84.6 | 86.7 |
| **Mean** | **64.75** | **71.2** | **82.8** | **84.7** | **84.85** | **85.85** |

2.3.2 Significance of difference in the glue yield

The glue yields in Table 4 are summarized in Table 5.

**Table 5. Glue yields of each treatment (unit: %)**

| Treatments | A  (30 min) | B  (60 min) | C  (90 min) | D  (120 min) | E  (150 min) | F  (180 min) | **Total** |
| --- | --- | --- | --- | --- | --- | --- | --- |
| 1 | 63.9 | 73.1 | 84.3 | 85.25 | 84 | 85.95 | 63.9 |
| 2 | 65.45 | 69.95 | 82.15 | 84.05 | 85.95 | 84.9 | 65.45 |
| 3 | 64.9 | 70.55 | 81.95 | 84.8 | 84.6 | 86.7 | 64.9 |
| ***T_i_*** | **194.25** | **213.6** | **248.4** | **254.1** | **254.55** | **257.55** | ***T* = 1422.45** |

1) Decomposition of the degrees of freedom and sums of squares

The degree of freedom of total variation = 6 × 3 –1 = 17

The degree of freedom between treatments = 6 – 1 = 5

The degree of freedom of error (within treatments) = 6 × (3 – 1) = 12

The correction factor *C* is calculated using variance analysis and various sums of squares are calculated:

The correction factor is *C* = = (1422.45)^2^ ÷ 18 = 112409.1112

The total sum of squares is SS_T_ = ∑*x*^2^ – *C* = 113599.6 – 112409.1112 = 1190.4888

The sum of squares between treatments is SS_t_ = – C = 340755.09 ÷ 3 – 112409.1112 = 1175.9188

The sum of squares of error (within treatments) is SS_e_ = SS_T_ – SS_t_ = 1190.4888 – 1175.9188 = 14.57

2) *F*-tests and variance analysis

The calculated results are listed in Table 6 and the *F*-value is calculated:

*F* = = 235.1838 ÷ 1.2142 = 193.6944

**Table 6. Variance analysis of data in Table 5**

| Sources of variation | DF | SS | MS | F | F_0.01_ |
| --- | --- | --- | --- | --- | --- |
| Between treatments | 5 | 1175.9188 | 235.1838 | 193.6944 | 5.06 |
| Error | 12 | 14.57 | 1.2142 |  |  |
| Total variation | 17 | 1190.4888 |  |  |  |

*F*_0.01_ is 5.06 (from tabulated values): because the *F* value (193.6944) is greater than *F*_0.01_ (5.06), it can be deduced that various treatments show significant differences in glue yields, so further multiple comparisons are needed for average values of each treatment.

3) Comparisons of average values of each treatment

Through new multiple-range tests, the standard error (SE) is calculated to be:

SE = = 0.6362

SSR_0.05_ and SSR_0.01_ values are taken from tabulated values of SSR at 5% and 1%. Using the following formula:

LSR_α_ = SE × SSR_α_

LSR_0.05_ and LSR_0.01_ values are calculated, as displayed in Table 7.

**Table 7. LSR values in new multiple-range tests of data in Table 5**

| ***P*** | **2** | **3** | **4** | **5** | **6** |
| --- | --- | --- | --- | --- | --- |
| SSR_0.05_ | 3.08 | 3.23 | 3.33 | 3.36 | 3.40 |
| SSR_0.01_ | 4.32 | 4.55 | 4.68 | 3.76 | 4.84 |
| LSR_0.05,12_ | 1.959 | 2.055 | 2.119 | 2.138 | 2.163 |
| LSR_0.01,12_ | 2.748 | 2.895 | 2.977 | 2.392 | 3.079 |

According to LSR_0.05_ and LSR_0.01_ scales in Table 7, multiple comparisons of the average glue yield of the six treatments (six different decoction durations) in Table 4 can be performed (Table 8).

**Table 8. Significance of difference in glue yields after six different decoction durations**

| Treatments (decoction durations) | Average glue yields | Significance of difference | |
| --- | --- | --- | --- |
|  |  | 5% | 1% |
| **F** (180 min) | 85.85 | **a** | **A** |
| **E** (150 min) | 84.85 | **ab** | **AB** |
| **D** (120 min) | 84.7 | **ab** | **AB** |
| **C** (90 min) | 82.8 | **b** | **B** |
| **B** (60 min) | 71.2 | **c** | **C** |
| **A** (30 min) | 64.75 | **d** | **D** |

According to results in Table 8, the glue yield of treatment D (120 min) differs insignificantly from that of treatment F (180 min) with the highest glue yield, however, treatment D saves about one-third of the decoction time compared with treatment F and therefore its power consumption is also reduced by about one-third compared with that of treatment F. Although treatment C (90 min) differs insignificantly from treatment D in terms of the glue yield, it shows significant differences with treatment F, reaching an *α*-level of 0.01. Therefore, analysis of the experimental results implies that treatment D is optimal with the highest efficiency ratio after comprehensive analysis and evaluation of the glue yield, time cost of decoction, and power consumption. That is, for the new glue-making technique designed in the research, 120 min (treatment D) is deemed to be the optimal decoction time.

3.3.2 Comparison of gluing strengths of the two glue-making techniques

(3) Significance of difference in gluing strength

1) Significance of difference in tensile shear strength

The tensile shear strengths in Table 12 are arranged in Table 14.

**Table 14. Tensile shear strengths of the two types of fish glue (unit: MPa)**

| Treatment | Traditional glue | New glue | Total |
| --- | --- | --- | --- |
| Specimen 1 | 5.26 | 8.26 |  |
| Specimen 2 | 5.70 | 8.23 |  |
| Specimen 3 | 9.37 | 7.13 |  |
| Specimen 4 | 9.43 | 8.87 |  |
| Specimen 5 | 8.40 | 5.93 |  |
| ***T_i_*** | **38.16** | **38.42** | ***T* = 76.58** |

1. Decomposition of the degrees of freedom and sums of squares

The degree of freedom of total variation = 2 × 5 – 1 = 9

The degree of freedom between treatments = 2 – 1 = 1

The degree of freedom of error (within treatments) = 2 × (5 – 1) = 8

The correction factor *C* is calculated using variance analysis and various sums of squares are calculated:

The correction factor is *C* = = (76.58)^2^ ÷ 10 = 586.4496

The total sum of squares is SS_T_ = ∑*x*^2^ – *C* = 608.0786 – 586.45 = 21.6286

The sum of squares between treatments is SS*_t_* = – *C* = 2932.282 ÷ 5 – 586.4496 = 0.0068

The sum of squares of error (within treatments) is SS_e_ = SS_T_ – SS_t_ = 21.6286 – 0.0068 = 21.6218

1. *F*-tests and variance analysis

The calculated results are listed in Table 15 and the *F*-value is calculated:

*F* = = 0.0068 ÷ 2.7027 = 0.0025

**Table 15. Variance analysis of data in Table 14**

| Sources of variation | DF | SS | MS | *F* | *F*_0.05_ | *F*_0.01_ |
| --- | --- | --- | --- | --- | --- | --- |
| Between treatments | 1 | 0.0068 | 0.0068 | 0.0025 | 5.32 | 11.26 |
| Error | 8 | 21.6218 | 2.7027 |  |  |  |
| Total variation | 9 | 21.63 |  |  |  |  |

It is tested that *F* (0.0025) is smaller than *F*_0.05_ (5.32), that is, it is insignificant in *F*-tests, so multiple comparisons between average values are not needed. This indicates that the fish glue prepared using the traditional handicraft does not differ significantly from that prepared using the new technique for assessing the tensile shear strength.

2) Significance of difference in compressive shear strength

The compressive shear strengths in Table 13 are arranged in Table 16.

**Table 16. Compressive shear strengths of the two types of fish glue (unit: MPa)**

| Treatment | Traditional glue | New glue | Total |
| --- | --- | --- | --- |
| Specimen 1 | 4.849 | 3.259 |  |
| Specimen 2 | 3.845 | 4.769 |  |
| Specimen 3 | 3.935 | 3.796 |  |
| Specimen 4 | 4.846 | 5.302 |  |
| Specimen 5 | 4.303 | 4.566 |  |
| ***T_i_*** | 21.778 | 21.692 | ***T* = 43.47** |

**①** Decomposition of the degrees of freedom and sums of squares

The degree of freedom (DOF) of total variation = 2 × 5 – 1 = 9

The DOF between treatments = 2 – 1 = 1

The DOF of error (within treatments) = 2 × (5 – 1) = 8

The correction factor *C* is calculated using variance analysis and various sums of squares are calculated:

The correction factor is *C* = = (43.47)^2^ ÷ 10 = 188.9641

The total sum of squares is SS_T_ = ∑*x*^2^ – *C* = 192.5142 – 188.9641 = 3.5501

The sum of squares between treatments is SS*_t_* = – *C* = 944.8242 ÷ 5 – 188.9641 = 0.0007

The sum of squares of error (within treatments) is SS_e_ = SS_T_ – SS*_t_* = 3.5501 – 0.0007 = 3.5494

② *F*-tests and variance analysis

The calculated results are listed in Table 17 and the *F*-value is calculated:

*F* = = 0.0007 ÷ 0.4437 = 0.0016

**Table 17.Variance analysis of data in Table 16**

| Sources of variation | DF | SS | MS | *F* | *F*_0.05_ | *F*_0.01_ |
| --- | --- | --- | --- | --- | --- | --- |
| Between treatments | 1 | 0.0007 | 0.0007 | 0.0016 | 5.32 | 11.26 |
| Error | 8 | 3.5494 | 0.4437 |  |  |  |
| Total variation | 9 | 3.5501 |  |  |  |  |

It is found that *F* (0.0016) is smaller than *F*_0.05_ (5.32), which indicates that it is insignificant, so multiple comparisons between average values are not needed. This suggests that the fish glue prepared using the traditional technique does not significantly differ from that prepared using the new technique for assessing the compressive shear strength.
